# Anatomical variations of the thoracic sympathetic ganglions and their effects on sympathicotomy for primary palmar hyperhidrosis

**DOI:** 10.1007/s10286-023-00932-2

**Published:** 2023-04-05

**Authors:** Guotian Pei, Shushi Meng, Yingshun Yang, Xiao Wang, Qiang Liu, Shuai Wang, Yuqing Huang

**Affiliations:** 1 Department of Thoracic Surgery, Beijing Haidian Hospital (Haidian Section of Peking University Third Hospital), No 29, Zhongguancun Street, Haidian District, Beijing, 100080 China; 2 Shanxi Key Laboratory of Artificial Intelligence-Assisted Diagnosis and Treatment for Mental Disorder, First Hospital of Shanxi Medical University, Shanxi, China

**Keywords:** Primary palmar hyperhidrosis, Sympathetic ganglion, Sympathicotomy, Near-infrared

## Abstract

**Purpose:**

The results and side effects of sympathicotomy for primary palmar hyperhidrosis (PPH) can vary due to anatomical variations of the sympathetic ganglions. The aim of our study was to clarify anatomical variations of the sympathetic ganglions by near-infrared (NIR) thoracoscopy and determine their effects on sympathicotomy for PPH.

**Methods:**

The cases of 695 consecutive patients with PPH treated with either R3 or R4 sympathicotomy either by normal thoracoscopy or by NIR fluorescent thoracoscopy between March 2015 and June 2021 were retrospectively reviewed and followed up.

**Results:**

The variation rate of third and fourth ganglions was 14.7% and 13.3% on the right side and 8.3% and 11.1% on the left side. Real T3 sympathicotomy (RTS_3_) was more effective than real T4 sympathicotomy (RTS_4_) in the short-term and long-term follow-up (*p* < 0.001 and *p* < 0.001, respectively). RTS_3_ was more satisfactory than RTS_4_ in the long-term follow-up (*p* = 0.03), but no significant difference was found in the short-term follow-up (*p* = 0.24). The incidence and severity of compensatory hyperhidrosis (CH) in the areas of the chest and back in the RTS_4_ group were significantly lower than those in the RTS_3_ group according to both the short-term results (12.92% vs. 26.19%, *p* < 0.001; 17.97% vs. 33.33%, *p* = 0.002, respectively) and the long-term results (19.66% vs. 28.57%, *p* = 0.017; 21.35% vs. 34.52%, *p* < 0.001, respectively).

**Conclusions:**

RTS_3_ may be more effective than RTS_4_ for PPH. However, RTS_4_ appears to be associated with a lower incidence and severity of CH in the areas of the chest and back than RTS_3._ NIR intraoperative imaging of thoracic sympathetic ganglions may improve the quality of sympathicotomy surgeries.

**Supplementary Information:**

The online version contains supplementary material available at 10.1007/s10286-023-00932-2.

## Introduction

Endoscopic thoracic sympathicotomy (ETS) has been accepted as the most effective method for the treatment of primary palmar hyperhidrosis (PPH), especially for those with a poor outcome from conservative treatments. The optimal level of sympathetic trunk interruption is the fibers between the second ganglion (G2; G refers to ganglion and the number refers to which ganglion) and third ganglion (G3) or between G3 and the fourth ganglion (G4), a procedure commonly known as T3 sympathicotomy or T4 sympathicotomy, respectively. However, sympathetic ganglions are usually obscured by fat and the pleura under normal thoracoscopy. Therefore, the ribs are frequently used as anatomical landmarks to indirectly determine the location of ganglia. In 2011, both the International Society of Sympathetic Surgery (ISSS) and the Society of Thoracic Surgeons (STS) proposed the use of rib-oriented nomenclature to describe the transected position on the sympathetic chain [1]. For example, if we cauterize the sympathetic chain on the fourth rib, which normally means that the sympathetic nerve is interrupted above the fourth ganglion (G4), this operation can be abbreviated as R4 sympathicotomy (R refers to the rib, and the number refers to which rib). Using this standardized nomenclature would allow for better communication among surgeons worldwide.

R3 or R4 sympathicotomy has been established as the standard approach for the treatment of PPH. However, the results and side effects of this procedure can vary due to anatomical variations of sympathetic ganglions; i.e., if the sympathetic ganglions are not in the normal position (a shift up or a shift down), then they will not represent the true level that needs to be interrupted. Previous studies demonstrated that the thoracic sympathetic ganglions can be visualized under near-infrared (NIR) fluorescent thoracoscopy combined with indocyanine green (ICG) [2], and the results from pathology studies confirmed their findings [3]. Also, our group subsequently confirmed that this novel technique is safe and feasible in ETS for PPH [4].

 The aim of the present study was to clarify the anatomical variations of the thoracic sympathetic ganglions by NIR thoracoscopy with ICG and determine their effects on sympathicotomy for PPH.

## Materials and methods

### Study population

The cases of 695 consecutive patients with PPH treated with either R3 or R4 sympathicotomy from March 2015 to June 2021, either by normal thoracoscopy or by NIR fluorescent thoracoscopy, were retrospectively reviewed and followed up. The inclusion criteria for patient enrollment were: (1) age between 18 and 75 years; (2) palmar sweating was the major complaint with or without axillary and plantar sweating; (3) no allergy to iodine or ICG for patients who underwent NIR fluorescent thoracoscopy. The exclusion criterion was presence of other diseases which may cause secondary hyperhidrosis. The choice of procedure performed (normal thoracoscopy or NIR fluorescent thoracoscopy) was based on the principle of voluntariness in which patients are provided with detailed information on these two surgical methods, including their specific advantages and disadvantages, and the patient then makes a choice and provides informed consent for the chosen surgical technique. For palmar-only hyperhidrosis, R3 or R4 interruption is recommended; for palmar and axillary hyperhidrosis, R3 + R5 or R4 + R5 interruption is recommended. Patients should be counseled on these surgical procedures, the surgical effects, the risks, and the side effects. Patients should be asked to participate in the decision-making process of the surgery.

 All operations were performed by the same surgical team.

 Approval for this study was waived by the Institutional Review Board.

### Defining R3/R4, T3/T4, G3/G4 sympathicotomy, and grouping

**Fig. 1:**
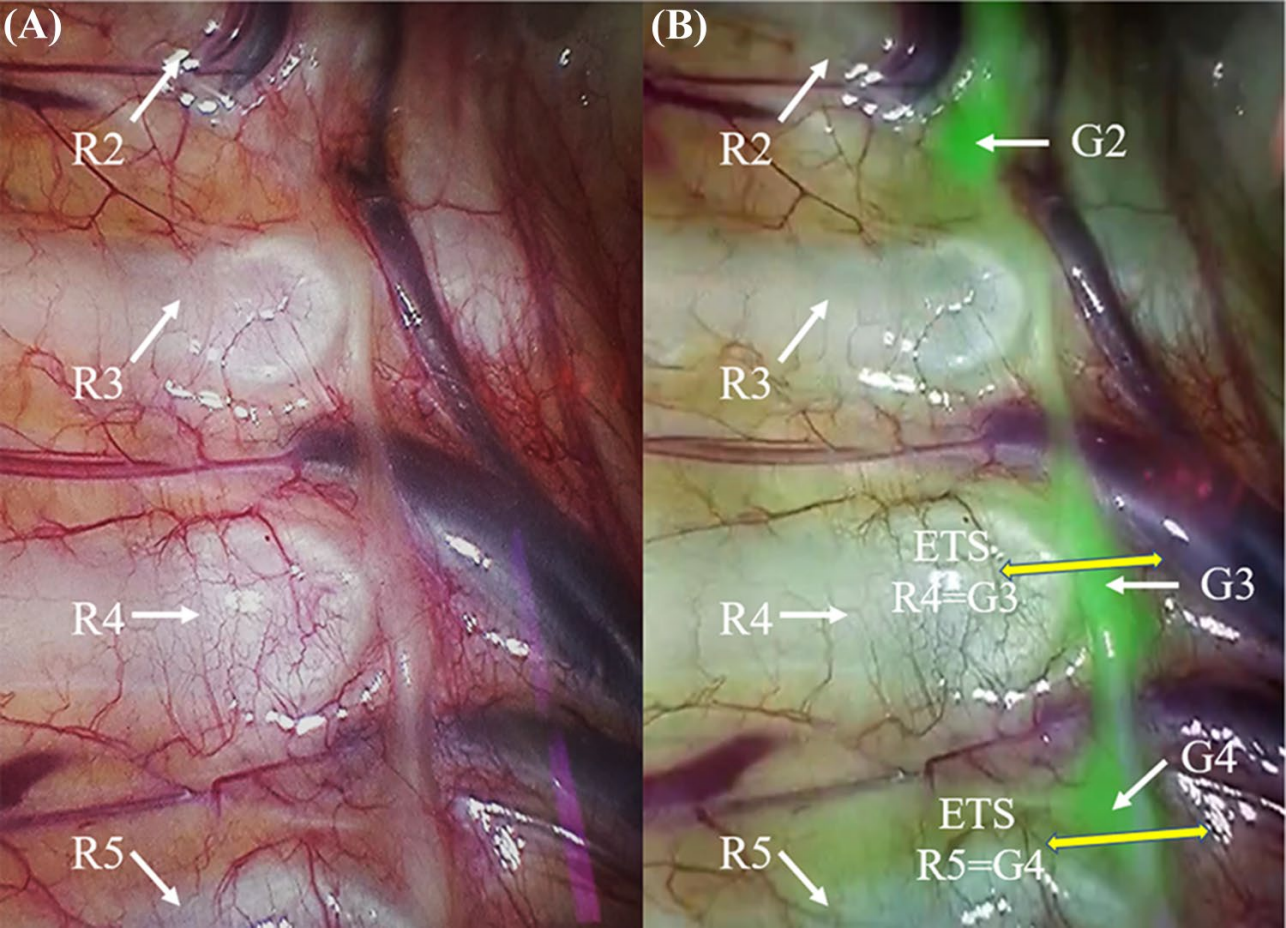
Sympathetic imaging by thoracoscopy. **a**,** b** Thoracoscopy in white light (**a**) and in near-infrared (NIR) light with indocyanine green (ICG) (**b**). The third (*G3*) and fourth (*G4*) ganglions can be seen to be shifted downward on the fourth and fifth rib (*R4* and* R5*, respectively); thus R4 = G3 sympathicotomy, and R5 = G4 sympathicotomy. *ETS* endoscopic thoracic sympathicotomy, G ganglion (with number referring to which ganglion), *R* rib (with number referring to which rib)

**Fig. 2:**
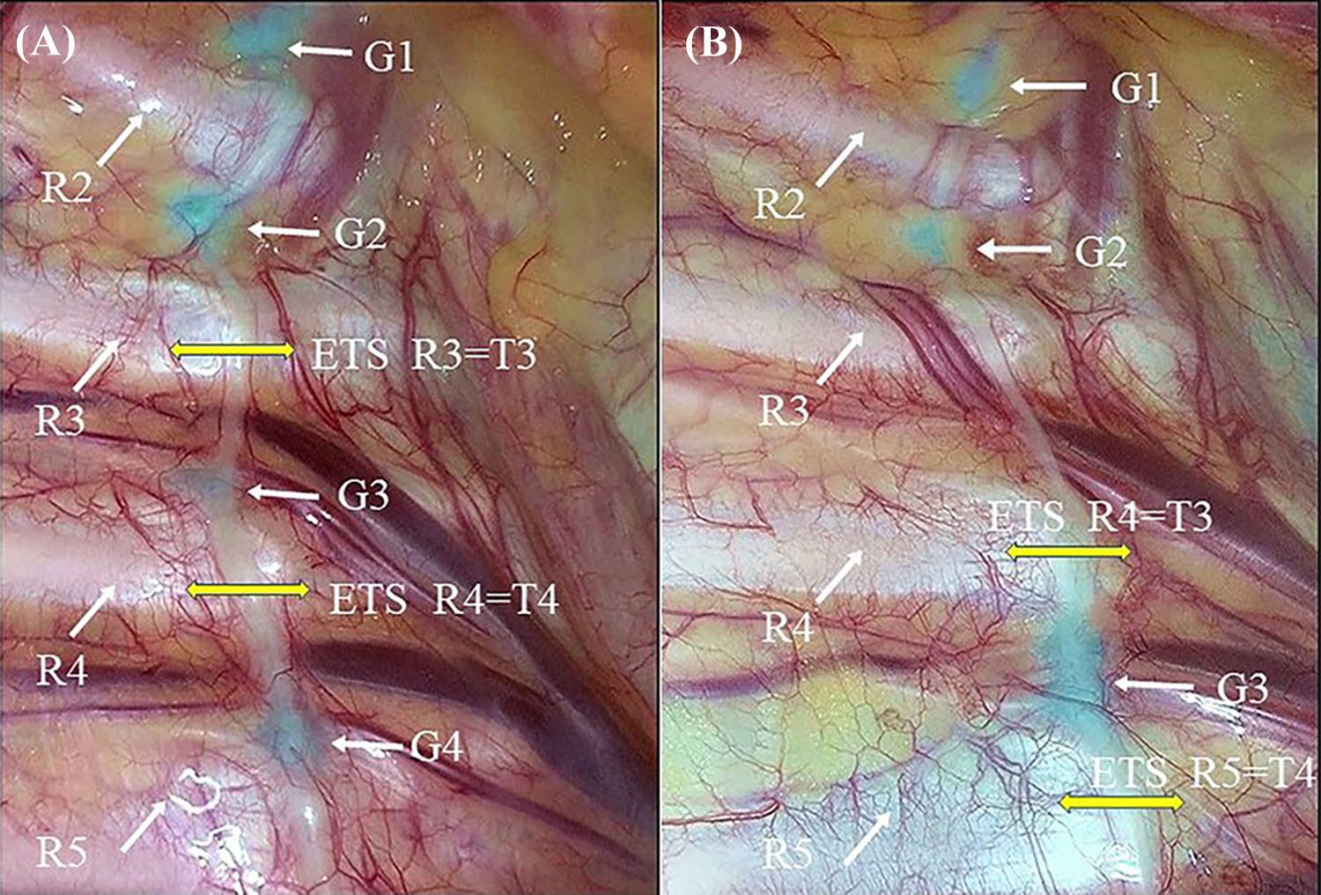
Sympathetic imaging by thoracoscopy. **a** Imaging in NIR light with ICG showing that all sympathetic ganglions are in the normal position. **b** Imaging in NIR light with ICG showing that G3 has shifted downward in the fourth intercostal space, thus R4 = T3 sympathicotomy, and R5 = T4 sympathicotomy

In NIR thoracoscopic surgeries, conventional R3 or R4 sympathicotomy was performed regardless of anatomical variations in the ganglions. Normal sympathetic ganglions were located in the corresponding intercostal space. If the sympathetic ganglions were not visible, we marked R3/R4 sympathicotomy as R3/R4 sympathicotomy. If the sympathetic ganglions were seen to be in a normal position, we marked R3/R4 sympathicotomy as T3/T4 sympathicotomy. If G3 had shifted downward onto the fourth rib, we marked R4 sympathicotomy as G3 sympathicotomy. If G4 had shifted downward onto the fifth rib, we marked R5 sympathicotomy as G4 sympathicotomy (Fig. 1). If G3 had shifted downward in the fourth intercostal space, we marked R4 sympathicotomy as T3 sympathicotomy. If G4 had shifted downward in the fifth intercostal space, we marked R5 sympathicotomy as T4 sympathicotomy (Fig. 2). Because no upward shift variation in G3 was identified on either side in the NIR thoracoscopic surgeries, we regarded R3 = T3 sympathicotomy in normal thoracoscopic surgeries.

Because the level of the blocked sympathetic signals is the same regardless of whether the procedure is performed by R3, G3, or T3 sympathicotomy, we classified R3, T3, and G3 sympathicotomy as real T3 sympathicotomy (RTS_3_). For the same reason, we classified G4 and T4 sympathicotomy as real T4 sympathicotomy (RTS_4_).

### Surgical procedure

For normal thoracoscopic surgeries and NIR thoracoscopic surgeries, ETS was performed as previously described [4, 5]. All procedures were performed by uniportal thoracoscopy under general anesthesia using a laryngeal mask airway. During the NIR thoracoscopic surgeries, the FloNavi™ Endoscopic Fluorescence Imaging System (Optomedic Technique Inc., Guangdong, China) was used to excite and capture the fluorescence signal of the ICG, resulting in the visualization of fluorescence and white light of the tissue. The sympathetic ganglions were recognized from top to bottom, and then the sympathetic chain was amputated by electrocautery regardless of whether the sympathetic ganglions were visible. The respective anatomy of G3 and G4 was recorded and analyzed during the operation. A chest radiograph was requested on the day of surgery to ensure complete lung expansion.

### Questionnaire

All patients with PPH needed to complete a preoperative questionnaire in the hospital, which included four sections: patient demographics, basic information on sweating, impact of sweating, and quality of life. Follow-up was completed using a postoperative questionnaire (including sections on improvement in sweating, impact of sweating, compensatory sweating and satisfaction) on the WeChat (Tencent Corp., Shenzhen, China) network using Sojump (Changsha ran Xing InfoTech Ltd., Changsha, China) within 3 months (short-term) and after 6 months (long-term) postoperatively. The first follow-up was completed for all patients within 3 months after surgery. The second follow-up was completed for all patients between 6 and 12 months after surgery and then followed up every 6 to 12 months. The last follow-up data were used for the long-term follow-up statistical analysis. The visual analog scale (VAS), with grades of between 1 (no sweating) and 4 (severe dripping sweat) was used to represent the sweating pattern. The degree of postoperative sweating was graded from VAS 1 (dry hands) to 4 (the same degree of sweating as before the surgery). The degree of compensatory hyperhidrosis (CH) after sympathicotomy was classified into the following four grades: (I) none; (II) mild, not bothersome; (III) moderate, bothersome but tolerable; and (IV) severe, intolerable, with the patient expressing regret for having undergone surgery. The degree of patient satisfaction after sympathicotomy was also graded using a VAS, from 1 (very dissatisfied) to 5 (very satisfied).

### Statistical analysis

Descriptive and inferential analyses were conducted using SAS version 9.4 software (SAS Institute, Cary, NC, USA). Continuous variables that met the assumption of normality were presented as the mean and standard deviation (age and body mass index [BMI]), while frequencies and percentages were used to describe categorical variables. *t*-tests were used to compare the differences in age and BMI between the RTS_3_ and RTS_4_ groups to determine the comparability of these two groups, and Fisher's exact test was used for assessment of gender effects. Wilcoxon rank-sum tests were conducted to test for differences in the efficacy and CH between the RTS_3_ and RTS_4_ groups. In addition, as efficacy, CH, and satisfaction were all rank variables, Spearman’s rank correlation coefficient was used to measure the association between these variables at two time points. Because the anatomical variations in the sympathetic ganglions might be different between the sides of the body, the reduction in palmar sweating between the two groups was compared manually. CH and patient satisfaction were compared based on number of patients. Significance levels were preset to* p* ≤ 0.05 based on two-tailed tests.

## Results

### Demographic data

A total of 440 patients with PPH (192 males and 248 females; all bilateral) underwent normal thoracoscopy, and 255 patients (133 males and 122 females; 254 bilateral and 1 unilateral procedure on the left side) underwent NIR fluorescent thoracoscopy. The patient who underwent unilateral surgery on the left side (R3 sympathicotomy) had undergone R3 sympathicotomy (right side) in Japan 5 months previously. Among our patients who underwent normal thoracoscopic surgeries, 14 underwent bilateral R3 sympathicotomy, 179 underwent bilateral R4 sympathicotomy, 21 underwent bilateral R3 + R5 sympathicotomy, and 218 underwent bilateral R4 + R5 sympathicotomy. Two patients in the R4 sympathicotomy group underwent repeat R3 sympathicotomy; the data on these two patients were not included in the data analysis. Eight individuals underwent different procedures on each side. Severe pleural adhesions and physical interference by either the aorta or adjacent intercostal vessels were the primary causes leading to bilateral differences in the procedures. In the NIR thoracoscopic surgeries, the sympathetic ganglions were observed on the right side in 218 patients (218/254, 85.83%) and on the left side in 216 patients (216/255, 84.71%). The true levels of interrupted sympathetic chains on each side are shown in Electronic Supplementary Material Table 1.

Because the sample of patients with RTS_3_ was small and no upward shift variation in G3 was identified on either side in the NIR thoracoscopic surgeries, we regarded R3 = T3 sympathicotomy in normal thoracoscopic surgeries. In total, 84 patients were treated with RTS_3_ and 178 patients were treated with RTS_4_. The two groups were comparable in terms of gender, age, and BMI (Table 1). All operations were successfully completed without any severe perioperative complications, and no procedures were converted to thoracotomy. Six patients exhibited a small unilateral or bilateral pneumothorax on follow-up chest X-ray, which resolved after conservative management. All patients were discharged 1–2 days after the operation. All patients in two groups completed the follow-up for 1.5–7 years.

**Table 1:** Characteristics of the two patient groups

Patient characteristics	Patient groups		*t*	*p* value
	RTS_3_ (*n* = 84)	RTS_4_(*n* = 174)		
Age (years)	26.21 ± 6.65	25.88 ± 5.94	0.40	0.69
BMI (kg/m^2^)	21.40 ± 2.72	21.49 ± 2.68	− 0.26	0.80
*Gender, n*				0.23
Male	35 (42%)	87 (50%)		
Female	49 (58%)	87 (50%)		

Values are presented as the mean ± standard deviation (SD), unless indicated otherwise

*BMI* Body mass index, *RTS*_*3*_ real T3 sympathicotomy, *RTS*_*4*_ real T4 sympathicotomy

### Anatomical variations of G3/G4 on each side

**Fig. 3:**
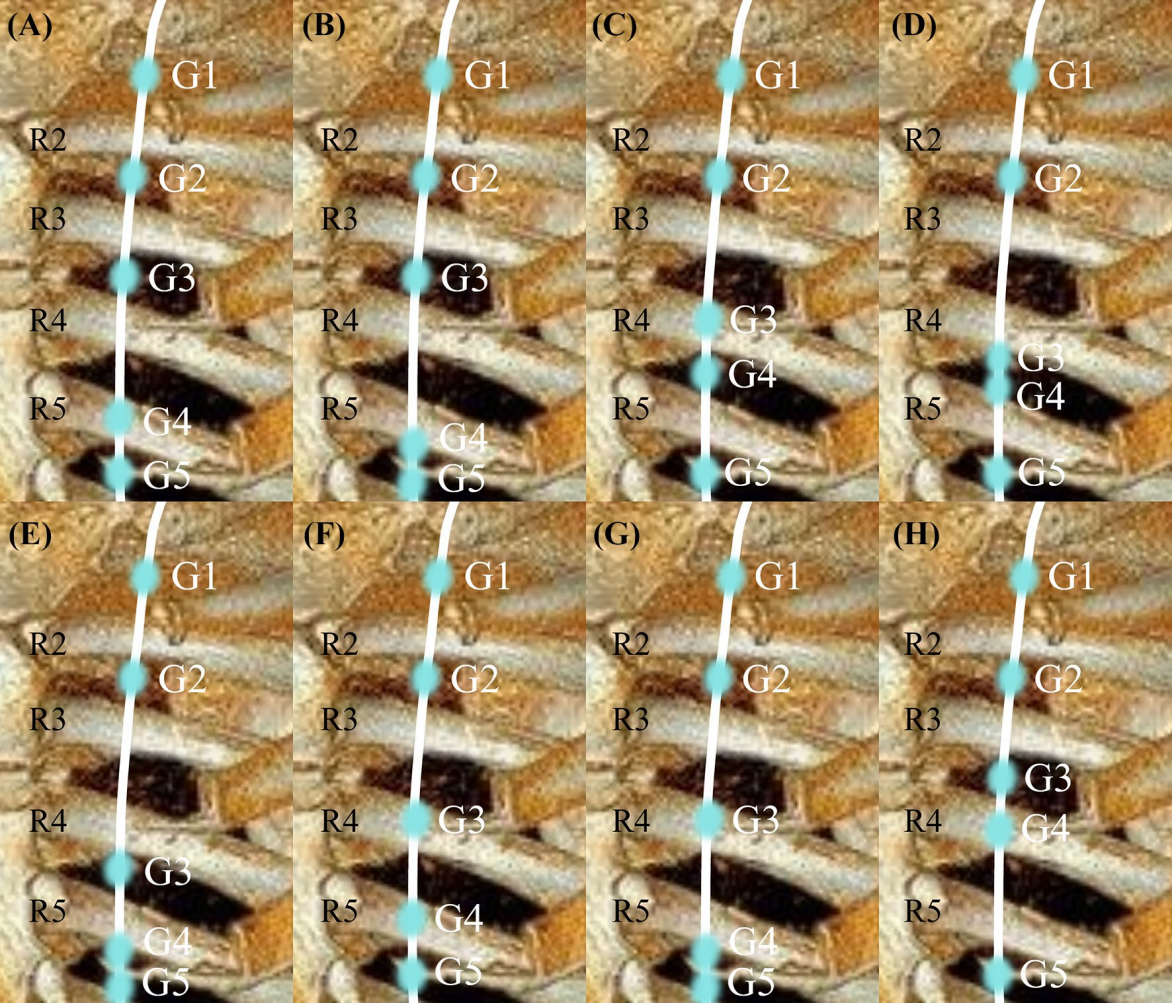
Simulation diagram for different types of variations of sympathetic ganglions. (**a**) G4 shifted downward on the fifth rib. (**b**) G4 shifted downward in the fifth intercostal space. (**c**) G3 shifted downward on the fourth rib. (**d**) G3 shifted downward in the fourth intercostal space. (**e**) G3 shifted downward in the fourth intercostal space and G4 shifted downward in the fifth intercostal space. (**f**) G3 shifted downward on the fourth rib and G4 shifted downward on the fifth rib. (**g**) G3 shifted downward on the fourth rib and G4 shifted downward in the fifth intercostal space. (**h**) G4 shifted upward on the fifth rib. R, rib; G, ganglion

Anatomical variations of G3/G4 were found in a total of 64 patients. Variations on the right side and left side were observed in 50 patients (22.9%, 50/218) and 38 patients (17.6%, 38/216), respectively. Bilateral variations were observed in 24 patients. No upward shift variation of G3 was identified on the right side, and no upward shift variation of G3 or G4 was identified on the left side. All results are shown in Table 2. We also identified a total of eight basic types of unilateral G3/G4 variations (Fig. 3).

**Table 2:** Anatomical variations in the position of the third and fourth ganglions on each side

Variations of G3/G4	Variation on the right side (*n* = 218)	Variation on the left side (*n* = 216)
*G3*	32 (14.7%)	18 (8.3%)
On the 4th rib	30 (13.8%)	16 (7.4%)
In the 4th intercostal space	2 (0.9%)	2 (0.9%)
*G4*	29 (13.3%)	24 (11.1%)
On the 4th rib	1(0.5%)	0
On the 5th rib	27 (12.4%)	22 (10.2%)
In the 5th intercostal space	1 (0.5%)	2 (0.9%)
*Concurrent variations of G3 and G4*	11 (5.0%)	4 (1.9%)
Total	50 (22.9%)	38 (17.6%)

Values in table are presented as a number (of patients) with the percentage in parentheses

*G* Ganglion (with the number indicating which ganglion)

### Symptom resolution

There was a significant difference in the resolution of palmar sweating (only accounting for nearly dry or dry hands) between the two groups (97.36% vs. 88.46%, *p* < 0.001) in the short-term follow-up. A significant difference in the resolution of palmar sweating was also found between the two groups (92.1% vs. 76.71%, *p* < 0.001) in the long-term follow-up. All results are shown in Table 3.

**Table 3:** Palmar sweating improvement in patients after endoscopic thoracic sympathicotomy in the short-term and long-term follow-up

Palmar sweating outcomes	Patient groups		*p* value
	RTS_3_	RTS_4_	
*Short-term follow-up*^a^	*n* = 152	*n* = 364	< 0.001
Dry hands	55 (36.18%)	170 (46.70%)	
Nearly dry hands (notable improvement)	93 (61.18%)	152 (41.76%)	
*Long-term follow-up*^a^	*n* = 152	*n* = 365	< 0.001
Dry hands	46 (30.26%)	109 (29.86%)	
Nearly dry hands (notable improvement)	94 (61.84%)	171 (46.85%)	

Values in table are presented as a number (of patients) with the percentage in parentheses

*ETS* Endoscopic thoracic sympathicotomy

^a^The reduction in palmar sweating between the two groups was compared manually

### Compensatory hyperhidrosis

The incidence and severity of different areas of CH after ETS in the short-term and long-term follow-up are shown in Table 4. The incidence of moderate-to-severe CH was significantly lower in the RTS_3_ group than in the RTS_4_ group (5.95% vs. 11.24%, *p* = 0.029) in the areas of the head and face in the short-term follow-up, but no significant difference was found during long-term follow-up (5.95% vs. 8.43%, *p* = 0.073). The incidence of moderate-to-severe CH was significantly lower in the RTS_4_ group than in the RTS_3_ group (17.41% vs. 28.57%, *p* = 0.039) in the areas of the legs in the long-term follow-up, but no significant difference was found in the short-term follow-up (17.42% vs. 23.81%, *p* = 0.842). The incidence of moderate-to-severe CH in the areas of the chest and back was significantly lower in the RTS_4_ group than in the RTS_3_ group in both the short-term (12.92% vs. 26.19%, *p* < 0.001; 17.97 vs. 33.33%, *p* = 0.002, respectively) and long-term follow-up (19.66% vs. 28.57%, *p* = 0.017; 21.35% vs. 34.52%, *p* < 0.001, respectively). No significant differences were found in the incidence and severity of CH in the areas of the waist, abdomen, buttocks, and feet.

**Table 4:** Incidence and severity of different areas of compensatory hyperhidrosis after endoscopic thoracic sympathicotomy in the short-term and long-term follow-up

Compensatory hyperhidrosis^a^	RTS_3_				RTS_4_				*p* value
	None	Mild	Moderate	Severe	None	Mild	Moderate	Severe	
*Short-term follow-up*									
Head and face	74 (88.10%)	5 (5.95%)	4 (4.76%)	1 (1.19%)	136 (76.40%)	22 (12.36%)	15 (8.43%)	5 (2.81%)	0.03*
Chest	43 (51.19%)	19 (22.62%)	17 (20.24%)	5 (5.95%)	132 (74.16%)	23 (12.92%)	18 (10.11%)	5 (2.81%)	< 0.001*
Back	29 (34.52%)	27 (32.14%)	21 (25.00%)	7 (8.33%)	94 (52.81%)	52 (29.21%)	24 (13.48%)	8 (4.49%)	0.002*
Waist	56 (66.67%)	12 (14.29%)	13 (15.48%)	3 (3.57%)	111 (62.36%)	41 (23.03%)	18 (10.11%)	8 (4.49%)	0.72
Abdomen	64 (76.19%)	8 (9.52%)	10 (11.90%)	2 (2.38%)	137 (76.97%)	24 (13.48%)	15 (8.43%)	2 (1.12%)	0.74
Buttocks	66 (78.57%)	8 (9.52%)	9 (10.71%)	1 (1.19%)	116 (65.17%)	42 (23.60%)	17 (9.55%)	3 (1.69%)	0.059
Legs	43 (51.19%)	21 (25.00%)	16 (19.05%)	4 (4.76%)	88 (49.44%)	59 (33.15%)	22 (12.36%)	9 (5.06%)	0.84
Feet	72 (85.71%)	5 (5.95%)	4 (4.76%)	3 (3.57%)	149 (83.71%)	21 (11.80%)	4 (2.25%)	4 (2.25%)	0.79
*Long-term follow-up*									
Head and face	74 (88.10%)	5 (5.95%)	4 (4.76%)	1 (1.19%)	140 (78.65%)	23 (12.92%)	10 (5.62%)	5 (2.81%)	0.073
Chest	40 (47.62%)	20 (23.81%)	18 (21.43%)	6 (7.14%)	113 (63.48%)	30 (16.85%)	28 (15.73%)	7 (3.93%)	0.017*
Back	27 (32.14%)	28 (33.33%)	20 (23.81%)	9 (10.71%)	98 (55.06%)	42 (23.60%)	28 (15.73%)	10 (5.62%)	< 0.001*
Waist	56 (66.67%)	13 (15.48%)	12 (14.29%)	3 (3.57%)	117 (65.73%)	31 (17.42%)	22 (12.36%)	8 (4.49%)	0.92
Abdomen	57 (67.86%)	14 (16.67%)	10 (11.90%)	3 (3.57%)	139 (78.09%)	18 (10.11%)	18 (10.11%)	3 (1.69%)	0.086
Buttocks	66 (78.57%)	8 (9.52%)	9 (10.71%)	1 (1.19%)	132 (74.16%)	25 (14.04%)	17(9.55%)	4(2.25%)	0.49
Legs	41 (48.81%)	19 (22.62%)	18 (21.43%)	6 (7.14%)	108 (60.67%)	39 (21.91%)	23 (12.92%)	8 (4.49%)	0.039*
Feet	70 (83.33%)	4 (4.76%)	5 (5.95%)	5 (5.95%)	148 (83.15%)	18 (10.11%)	8 (4.49%)	4 (2.25%)	0.87

Values in table are presented as a number (of patients) with the percentage in parentheses

*p ≤ 0.05, indicating a significant difference in compensatory hyperhidrosis in the specified area of the body between the two groups

^a^Compensatory hyperhidrosis was compared by the number of patients

### Patient satisfaction

Patient satisfaction over the short term and long term after ETS in the two groups is shown in Table 5. Patient satisfaction was significantly higher in the RTS_3_ group than in the RTS_4_ group (*p* = 0.03) in the long-term follow-up, but no significant difference was found in the short-term follow-up (*p* = 0.24). The main reasons for dissatisfaction were severe CH in the RTS_3_ group and recurrence in the RTS_4_ group. Our analysis of factors influencing satisfaction are shown in Table 6. Short-term satisfaction was directly related to short-term efficacy (reduction in palmar sweating; *p* < 0.0001) and the regions of CH in the areas of the head and face (*p* = 0.004), chest (*p* < 0.0001), back (*p* < 0.0001), waist (*p* < 0.0001), abdomen (*p* = 0.001), buttocks (*p* < 0.0001), legs (*p* < 0.0001), and feet (*p* = 0.002). Long-term satisfaction was directly related to long-term efficacy (reduction in palmar sweating; *p* < 0.0001) and the regions of CH in the areas of the chest (*p* < 0.0001), back (*p* < 0.0001), waist (*p* = 0.007), buttocks (*p* < 0.001), legs (*p* = 0.003), and feet (*p* < 0.0001).

**Table 5:** Patient satisfaction after endoscopic thoracic sympathicotomy over the short term and long term

Patient satisfaction^a^	RTS_3_	RTS_4_	*P* value
*Short-term satisfaction*	*n* = 84	*n* = 174	0.24
Very dissatisfied	0 (0.00%)	3 (1.72%)	
Dissatisfied	1 (1.19%)	5 (2.87%)	
Moderate	13 (15.48%)	19 (10.92%)	
Satisfied	25 (29.76%)	71 (40.80%)	
Very satisfied	45 (53.57%)	76 (43.68%)	
*Long-term satisfaction*	*n* = 80	*n* = 166	0.03
Very dissatisfied	0 (0.00%)	5 (2.98%)	
Dissatisfied	2 (2.44%)	6 (3.57%)	
Moderate	10 (12.20%)	28 (16.67%)	
Satisfied	29 (35.37%)	72 (42.86%)	
Very satisfied	39 (47.56%)	55 (32.74%)	

Values in table are presented as a number (of patients) with the percentage in parentheses

^a^Patient satisfaction was compared based on the number of patients

**Table 6:** Spearman’s rank correlation coefficient between satisfaction and efficacy or compensatory hyperhidrosis

Variables	Patient satisfaction according to follow-up duration			
	Short-term follow-up		Long-term follow-up	
	Correlation coefficient	*p* value	Correlation coefficient	*p* value
*Palmar sweating*^a^				
Left hand	− 0.262	< 0.001	− 0.148	< 0.001
Right hand	− 0.254	< 0.001	− 0.150	< 0.001
*Regions of CH*				
Head and face	− 0.109	0.004	− 0.046	0.23
Chest	− 0.187	< 0.001	− 0.179	< 0.001
Back	− 0.219	< 0.001	− 0.165	< 0.001
Waist	− 0.195	< 0.001	− 0.104	0.007
Abdomen	− 0.122	0.001	− 0.029	0.44
Buttocks	− 0.156	< 0.001	− 0.124	0.001
Legs	− 0.170	< 0.001	− 0.113	0.003
Feet	− 0.120	0.002	− 0.173	< 0.001

*CH* Compensatory hyperhidrosis

^a^Palmar sweating, degrees of palmar sweating after surgery

## Discussion

Endoscopic thoracic sympathicotomy has been proven to be a safe and most effective treatment for PPH, with R3 or R4 sympathicotomy being established as the standard approach for palmar hyperhidrosis. However, the results and side effects of this procedure can vary due to anatomical variations of sympathetic ganglions [6–9].The sympathetic ganglions are usually obscured by fat and the pleura under normal thoracoscopy. Therefore, ribs are frequently (only) used as anatomical landmarks to indirectly determine the location of ganglia. To address this issue, previous studies reported NIR fluorescent imaging of the sympathetic ganglion and confirmed that the fluorescence signal comes from neurocytes in the ganglion under fluorescence microscopy [2, 3]. This novel technique was later proven safe and effective in ETS [4]. Because the relationship between the anatomical variation in sympathetic ganglions and the surgical outcomes is not clear, we only observed and recorded the location of the sympathetic ganglions during the operation; that is, the imaging results of the sympathetic ganglions during the operation did not guide the choice of surgical procedure. In this context, we conducted this retrospective study to clarify the anatomical variations of the thoracic sympathetic ganglions and determine their effects on sympathicotomy for PPH.

To our knowledge, this study enrolled the largest sample size to date compared to previous studies on anatomical variations of G3 and G4 by NIR thoracoscope in patients with PPH. Most of the previous studies on thoracic sympathetic variation were based on human cadavers, with a limited sample size for dissection [10, 11]. In the present study, the anatomical variation rate of G3/G4 was higher on the right side (22.9%) than on the left side (17.6%). A considerable number of patients with PPH had bilateral variations (*n* = 24), and it is known that the type of variation may be different on each side. Variations of both G3 and G4 on bilateral sides could be identified simultaneously in three patients. We found eight basic types of unilateral G3/G4 variations, which could be described in more detail than in previous studies by using NIR thoracoscopy with ICG. A wide variety of G3/G4 variations were present in 64 patients, and such variations could have affected the postoperative results in some patients with PPH.

In terms of the therapeutic effect, the results of all previous case–control studies and meta-analyses of R(T)3 and R(T)4 sympathicotomy indicated that both procedures have good curative effects. RT_4_ sympathicotomy was associated with a lower incidence of CH than RT_3_ sympathicotomy [12–15]. These procedures were performed by normal thoracoscopy and the sympathetic ganglions were not visible. If G3 shifted down to the fourth rib or the fourth intercostal space, conventional R4 sympathicotomy would in fact be G3 or T3 sympathicotomy, which could be a potential confounding factor influencing the results. In our study, there was a significant difference in symptom resolution between the two groups (97.37% vs. 88.46%, *p* < 0.001) in the short-term follow-up, and a significant difference was also found between the two groups (92.11% vs. 76.71%, *p* < 0.001) in the long-term follow-up although 14 patients (3.85%) in the RTS_4_ group relapsed during long-term follow-up. Weng et al. also reported that 15 patients who underwent R4 ± R5 sympathicotomy relapsed within the first 2 years after surgery [5], indicating that the therapeutic effect could decrease slightly over time [16–18] and highlighting the risk of recurrence after R4 ± R5 sympathicotomy.

CH is the most common side effect—not complication—of ETS and greatly affects patient satisfaction. The reported incidence of CH varies from 3% to 98% [19–23]. CH occurs most frequently in the areas of the back and abdomen [21], and most patients present with two or more affected areas [24, 25]. Most past studies have focused on the incidence and severity of CH after ETS between R(T)3 and R(T)4 sympathicotomy [19, 26], rather than the areas and severity of CH between the two groups. In our study, we compared the incidence and severity of different areas of CH after ETS between RTS_3_ and RTS_4_ in the short-term and long-term follow-up. In the short-term follow-up, the overall incidence of CH in the areas of the head and face was 11.9% in the RTS_3_ group and 23.6% in the RTS_4_ group, which can be interpreted as follows: for palmar-only hyperhidrosis, the incidence of CH in the areas of the head and face was significantly lower in the RTS_3_ group than in the RTS_4_ group in the short-term results; for palmar and craniofacial hyperhidrosis, craniofacial sweating was effectively alleviated after RTS_3_ sympathicotomy than RTS_4_ sympathicotomy in the short-term results. Regarding this point, in 2011 the STS also proposed the R3-alone interruption for craniofacial hyperhidrosis [1]. The incidence of both overall CH and moderate-to-severe CH in the areas of the chest and back was significantly higher in the RTS_3_ group than in the RTS_4_ group. No significant differences were found in the incidence and severity of CH in the areas of the waist, abdomen, buttocks, and feet. The above results suggest that RTS_4_ is associated with a lower incidence of CH in the areas of chest and back than RTS_3_. Several authors have reported that CH is greatly reduced over time and stabilizes at 6 months after surgery [20, 27]. Herbst et al. also reported that 67.4% of their patients still had CH after a follow-up period of 14.6 years [28]. Based on our results, it must be admitted that the notion that CH will vanish over time is probably incorrect.

Patient satisfaction is related not only to the reduction in palmar sweating but also to the incidence and severity of CH [29]. In the present study, patient satisfaction was similar (*p* = 0.24) between the two groups in the short-term follow-up. However, RTS_3_ was assessed to be more satisfactory than RTS_4_ (*p* = 0.03) in the long-term follow-up. Therefore, the level of patient satisfaction will drop if the therapeutic effect is reduced during the long-term follow-up. For CH, patient satisfaction was closely related to the regions of CH both in the short-term and long-term follow-up in our study. In previous studies, CH was a complaint that increased in importance over time [28]; once the euphoria over improvement in their palmar sweating dissipated, the patient’s dissatisfaction with CH increased [29]. Therefore, thorough and effective communication with patients before surgery to inform them of the incidence of CH is important to increase the satisfaction rate.

The results of this study should be interpreted with care, keeping several limitations in mind. The major limitation was its non-randomized design. Therefore, unknown confounding variables could have biased the results. Second, although there was no upward shift variation in G3 in our NIR thoracoscopic surgeries, this did not mean that G3 of all patients with PPH did not shift upward. This might have affected the comparability of the data. However, despite these limitations, to our knowledge, this study is the first to compare the results of RTS_3_ and RTS_4_ for PPH.

We conclude that RTS_3_ may be more effective than RTS_4_ for PPH both in the short-term follow-up and long-term follow-up. RTS_3_ is more satisfactory than RTS_4_ in terms of long-term outcomes. However, RTS_4_ appears to be associated with a lower incidence of overall CH and moderate-to-severe CH in the areas of the chest and back than RTS_3_ according to the results of both the short-term and long-term follow-up. Patients should be aware of these differences and encouraged to participate in the decision-making process regarding the intended procedure. NIR intraoperative imaging of thoracic sympathetic ganglions may improve the quality of sympathicotomy surgeries. Future randomized controlled studies are needed to confirm this conclusion.

## Supplementary Information

Below is the link to the electronic supplementary material.

Supplementary file1 (DOCX 14 KB)

## Data Availability

The data that support the findings of this study are available on request from the corresponding author.

## Declarations

### Prior presentation

The abstract was presented as an e-poster at the Society of Thoracic Surgeons (STS) 2022 (abstract title: Anatomical Variations of the Thoracic Sympathetic Ganglions by Near-Infrared Thoracoscope with Indocyanine Green) and presented as an oral presentation at the 14th International Symposium on Sympathetic Surgery of the ISSS (abstract title: Real T3 vs T4 endoscopic thoracic sympathicotomy for primary palmar hyperhidrosis).
