# Supplementary material for: Anatomical variations of the thoracic sympathetic ganglions and their effects on sympathicotomy for primary palmar hyperhidrosis
Source: Clin Auton Res. 2023 Apr 5;33(2):111–20. doi: 10.1007/s10286-023-00932-2 (PMC10182923; doi:10.1007/s10286-023-00932-2)
Supplement: Supplementary file 1 — Supplementary file1 (DOCX 14 KB) [file 10286_2023_932_MOESM1_ESM.docx]

Supplementary Table 1. The True Levels of Interrupted Sympathetic Chains on Each Side in NIR Thoracoscopic Surgeries.

| Level of sympathicotomy | Right side | Left side |
| --- | --- | --- |
| R3 sympathicotomy | n=8 | n=8 |
| R3=R3 sympathicotomy | 2 | 0 |
| R3=T3 sympathicotomy | 6 | 8 |
| R3+R5 sympathicotomy | n=2 | n=2 |
| R3+R5=T3+T5 sympathicotomy | 2 | 2 |
| R4 sympathicotomy | n=81 | n=84 |
| R4=R4 sympathicotomy | 10 | 12 |
| R4=G3 sympathicotomy | 13 | 5 |
| R4=G4 sympathicotomy | 1 | 0 |
| R4=T4 sympathicotomy | 57 | 67 |
| R4+R5 sympathicotomy | n=163 | n=161 |
| R4+R5=R4+R5 sympathicotomy | 24 | 27 |
| R4+R5=G3+G4 sympathicotomy | 11 | 4 |
| R4+R5=G3+T5 sympathicotomy | 6 | 7 |
| R4+R5=T3+T5 sympathicotomy | 2 | 2 |
| R4+R5=G4+T4 sympathicotomy | 16 | 18 |
| R4+R5=T4 sympathicotomy | 1 | 2 |
| R4+R5=T4+T5 sympathicotomy | 103 | 101 |
| Total | 254 | 255 |

NIR, near-infrared, R, rib; G, ganglion.
